# Spatiotemporal Variation and the Role of Wildlife in Seasonal Water Quality Declines in the Chobe River, Botswana

**DOI:** 10.1371/journal.pone.0139936

**Published:** 2015-10-13

**Authors:** J. Tyler Fox, Kathleen A. Alexander

**Affiliations:** 1 Department of Fish and Wildlife Conservation, Virginia Polytechnic Institute and State University, Blacksburg, Virginia, United States of America; 2 CARACAL: Centre for Conservation of African Resources, Kasane, Botswana; University of Minnesota, UNITED STATES

## Abstract

Sustainable management of dryland river systems is often complicated by extreme variability of precipitation in time and space, especially across large catchment areas. Understanding regional water quality changes in southern African dryland rivers and wetland systems is especially important because of their high subsistence value and provision of ecosystem services essential to both public and animal health. We quantified seasonal variation of *Escherichia coli (E*. *coli*) and Total Suspended Solids (TSS) in the Chobe River using spatiotemporal and geostatistical modeling of water quality time series data collected along a transect spanning a mosaic of protected, urban, and developing urban land use. We found significant relationships in the dry season between *E*. *coli* concentrations and protected land use (p = 0.0009), floodplain habitat (p = 0.016), and fecal counts from elephant (p = 0.017) and other wildlife (p = 0.001). Dry season fecal loading by both elephant (p = 0.029) and other wildlife (p = 0.006) was also an important predictor of early wet season *E*. *coli* concentrations. Locations of high *E*. *coli* concentrations likewise showed close spatial agreement with estimates of wildlife biomass derived from aerial survey data. In contrast to the dry season, wet season bacterial water quality patterns were associated only with TSS (p<0.0001), suggesting storm water and sediment runoff significantly influence *E*. *coli* loads. Our data suggest that wildlife populations, and elephants in particular, can significantly modify river water quality patterns. Loss of habitat and limitation of wildlife access to perennial rivers and floodplains in water-restricted regions may increase the impact of species on surface water resources. Our findings have important implications to land use planning in southern Africa’s dryland river ecosystems.

## Introduction

In water-stressed regions like southern Africa freshwater resources are under increasing extractive pressures that complicate sustainable management of these systems. Semi-arid and arid regions together constitute nearly 50% of global land area while supporting approximately 20% of the global population [1, 2]. Rivers in dryland regions exhibit a diversity of forms and behaviors, but typically have greater flow variability than their tropical and temperate counterparts [3, 4]. Precipitation in dryland river basins is exceeded by evapotranspiration, and rainfall is extremely variable in time and space, especially across moderate to large catchments (>100 km^2^) where both intensity and runoff tend to be high [5]. Seasonal flood pulses in these dryland systems can have significant impacts on the timing of agriculture and other human landscape uses, as well as on the movement and ecology of native wildlife [6, 7].

Limited infrastructure and uneven temporal and spatial distribution of clean water resources in southern Africa, a predominantly dryland region, means only 61% of the region’s population has reliable access to safe drinking water [8]. Frequent disruptions to existing water treatment and delivery systems can have significant impacts on health and prosperity of human populations [9]. Diarrheal disease is the leading cause of child malnutrition worldwide and the second leading cause of mortality for children under five years of age, with nearly half of all diarrheal deaths occurring in sub-Saharan Africa [10]. The long-term effects of persistent and repeated bouts of diarrheal disease caused by waterborne pathogens such as *Escherichia coli (E*. *coli)* can be severe, with infections resulting in significant cognitive and physical development problems that can have significant life time effects [11–17].

Perennial dryland rivers in southern Africa and their associated wetlands often provide a major proportion of water for drinking and irrigation, as well as food resources, traditional medicines, roof thatching, and other materials for building and crafts [18]. Access to clean water is a critical ecosystem service is fundamental to human health, and while surface water resources are limited and of critical importance to dryland regions, land areas abutting surface water are of high value and often a focus of human development. Prioritization of waterfront access for human development can restrict and compress wildlife populations particularly in Africa into increasingly smaller areas where routes to surface water remain open. While concentrated wildlife can be a boon for ecotourism operations, substantial environmental impacts may ensue, including loss or modification of woody vegetation structure and cover, soil erosion, and increased rates of fecal deposition [19]. Heavy rainfall and seasonal flooding in impacted areas may amplify surface water inputs of fecal bacterial contamination from water and sanitation system overflows, and runoff of fecal material and sediment from the surrounding landscape [20, 21]. Elevated bacterial concentrations in river courses may arise through exogenous sources including fecal loading by humans, livestock, and wildlife concentrated around limited surface water resources, or through microbial survival and growth in-situ [20, 22–25].

Evaluation of water quality and sustainability of southern Africa’s perennial rivers is particularly important given future regional climate change scenarios, which predict a 10–30% decrease in runoff to occur in southern Africa by 2050 [26]. Even a 10% drop in long-term average annual rainfall amounts in dryland countries such as Botswana in southern Africa is predicted to result in a minimum 42% reduction of perennial drainage entering the country’s rivers [27]. With 94% of Botswana’s water resources shared among neighboring countries [28], sustainable conservation of perennial surface water sources will have considerable consequences for the region’s economies, ecosystems, and associated communities. In this study, we use the dryland Chobe River system in Northern Botswana to evaluate the interaction between season, land use, and wildlife and the influence this can have on the spatial and temporal dynamics of water quality in a dryland system.

## Materials and Methods

### Study Site

The Chobe River in northeast Botswana is part of the vast and interconnected Okavango-Kwando-Zambezi catchment system (~693,000 km^2^), which has its headwaters in the highlands of Angola [29]. This semi-arid, subtropical region is characterized by highly variable seasonal rainfall, with nearly all the annual precipitation (avg. 604 mm) occurring during the summer wet season (December–April), with a general absence of precipitation during the dry season (May–November). The Chobe River is one of three permanent surface water sources in Botswana and the only permanent surface water source in the entire Chobe District (21,000 km^2^). Settlements in the Chobe District relied almost entirely upon drinking water supplies abstracted from the Chobe River from a single municipal water intake and treatment facility located in Kasane at the time data were collected [9]. Land use within the Chobe District includes communal and private lands used for grazing livestock and agriculture, protected land including forest reserves and wildlife management areas, as well as villages and urban townships. A dominant feature of the study area is the Chobe National Park (CNP), Botswana’s second largest (11,700 km^2^), which provides critical habitat for the largest elephant (*Loxodonta africana)* population in Africa, as well as a host of other wildlife species. Kasane is the largest urban area in Chobe District and the seat of local government with an estimated population of 9,008 [30]. Kazungula is a growing residential and commercial area (est. population 4,133), as well as a border crossing to Zambia by ferry and Zimbabwe by road.

#### Chobe River floodplain

**Fig 1:**
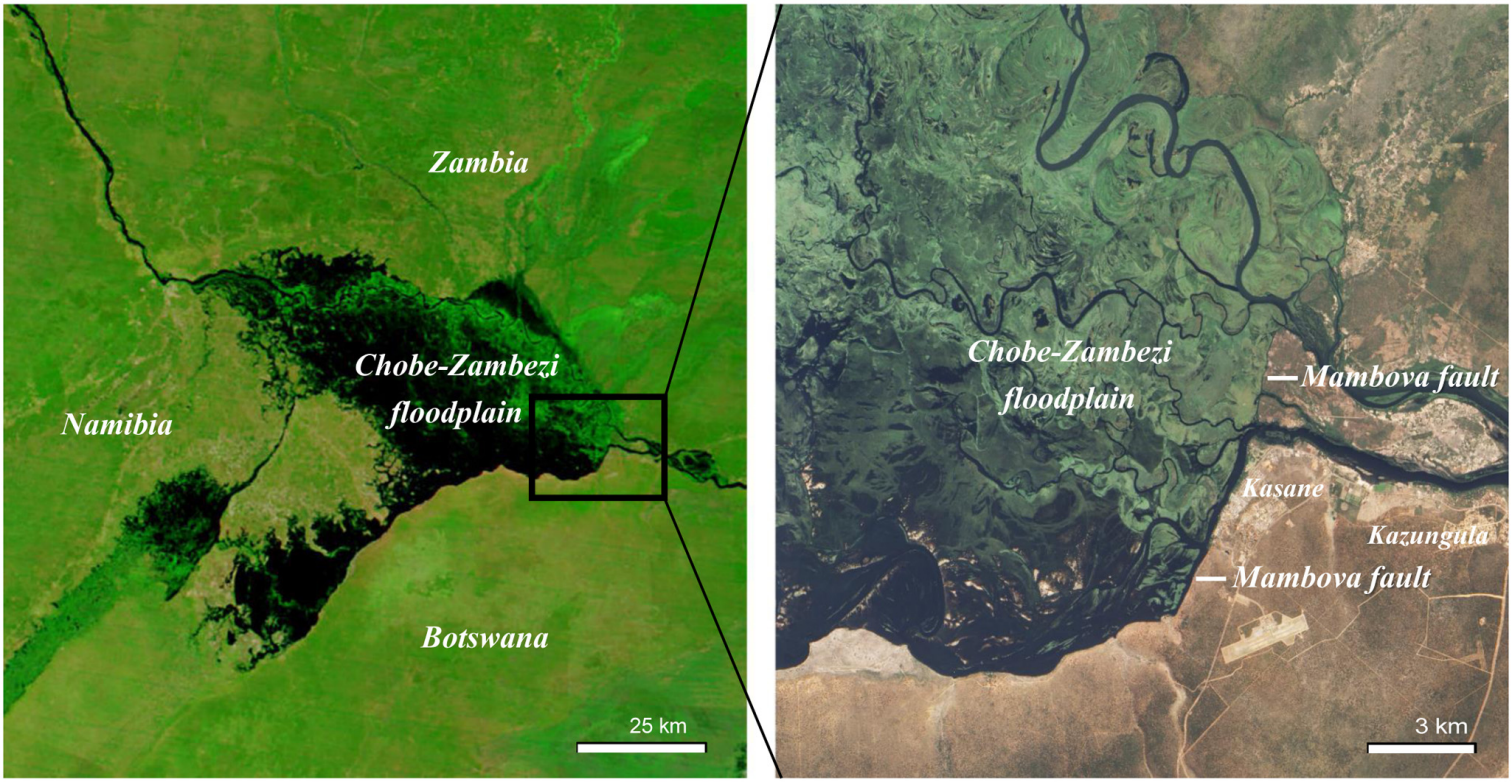
MODIS Terra (left) and Advanced Land Imager, EO-1 satellite imagery acquired on same day, May 8, 2010 (data available from the U.S. Geological Survey). The vast Chobe-Zambezi floodplain system is visible at two different scales showing the dominant geological and hydrological influence of the Mambova fault.

Soils of the Chobe River’s floodplain and alluvial terrace are fine textured and relatively nutrient-rich, with a generally high clay content that increases with depth [31]. While generally buried at depth by well-drained, nutrient-poor Kalahari quartz sand across the greater proportion of the study area, basalts of the Stormberg lava origin become particularly exposed on the northern rim of the Chobe Plateau above Kasane. The plateau face is generally steepest (990 meters a.s.l) near the confluence with the Zambezi River and approaches closest to the Chobe River in the town of Kasane, which is itself perched several meters above the river on the steep edge of the plateau. The Chobe River channel widens considerably as the river flows east through the CNP, before narrowing and deepening as it cuts across the Mambova fault and joins the Zambezi River. The relatively active fault line defines the eastern edge of the Chobe River floodplain and is a dominant geological feature controlling the location and hydrological character of the present-day Chobe River channel [32]. During the annual flood, water builds up behind the fault line and spreads outwards across the river floodplain, reaching as far upstream as Lake Liambezi in high flood years (Fig 1).

#### Chobe River transect

**Fig 2:**
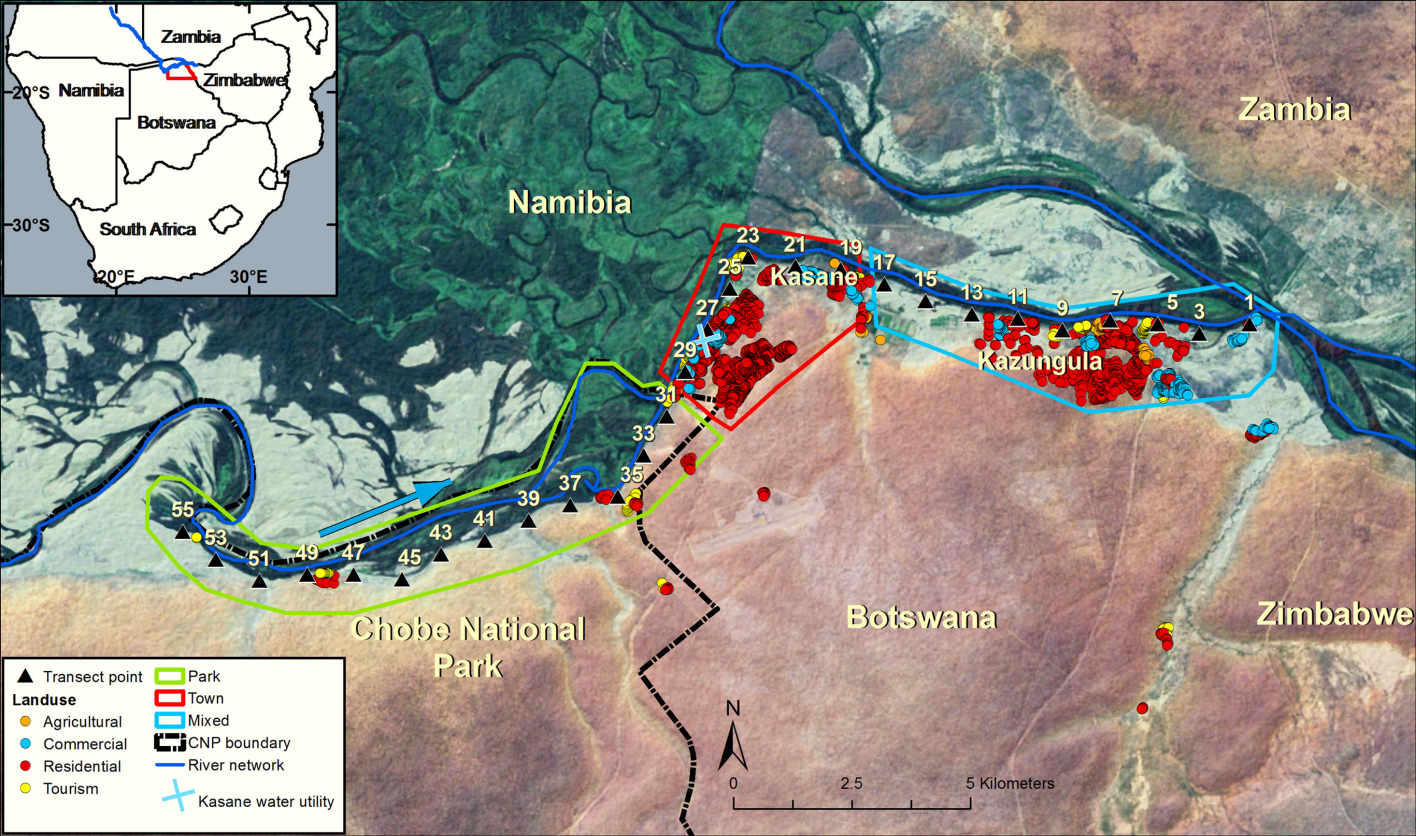
Landsat-based map of the Chobe River study area. Dominant land uses are shown within the three representative land use types, which include: Park (solid green line), Town (red), and Mixed-use (light blue). The Chobe National Park border is also shown (dashed black line), along with water quality transect points (black triangles) and the Kasane water purification facility (blue cross). Chobe River flow direction is indicated by the blue arrow.

We established a transect consisting of 55 points with 500m spacing located along a 27.5 km reach of the Chobe River (Fig 2), starting at the confluence of the Chobe and Zambezi Rivers (transect 1: 17°47’41.4854”S, 25°15’38.9874”E) and ending approximately 12 km into the CNP (transect 55: 17°49’56.928”S, 25°2’52.7274”E). Land use along the Chobe River transect from west (upstream) to east (downstream) consisted of: Park (transect points 55–31), Town (29–19), and Mixed (17–1). Kasane and Kazungula are located within Town and Mixed land use areas, respectively. Town land use includes residential and commercial areas, as well as various tourist facilities. The CNP is a primary ecotourism destination. Annual visitation to the park increased from just over 60,000 in 1995 to more than 110,000 in 2004, with the majority of visitation occurring during the dry season [33]. Human settlements are absent within the CNP and riverfront and riparian habitats are heavily utilized by water-dependent wildlife, particularly during the dry season when alternative food and water sources are limited [7]. Over the past forty years, elephant populations within the CNP in have grown at a mean annual rate of between 5.5% and 7% and currently are among the largest on the African continent [34, 35]. Two small commercial farms are located in the mixed land use area, which grow fruits and vegetables for local sale. Extensive farming is severely constrained by crop raiding and destruction by wildlife, and livestock production systems are focused on subsistence livelihoods. Cattle densities in the study area are have been low since the spread of tsetse fly (*Glossina spp*.) forced the gradual abandonment of cattle posts after the 1940s, and endemic diseases like Foot-and-mouth disease (*Aphthae epizooticae*) prevent the sale of animals to the Botswana Meat Commission [30].

### Water Sample Collection and Analysis

Collection of grab samples was conducted bi-monthly from July 7, 2011 to March 19, 2014 (n = 1630) at 1km intervals across the three land use classes (S1 Table). Samples were taken approximately ten meters from the south (Botswana) riverbank from just below the water surface. Abiotic water quality data including dissolved oxygen (DO, mg/L), specific conductivity (Cond, mS/cm), and water temperature (°C) were measured from April 25, 2012 –January 31, 2013 (n = 512) using a YSI Pro2030 (YSI Inc., Yellow Springs, Ohio, USA), concurrent with water sample collection. Collection of abiotic water quality data for the entire sampling period was limited due to equipment failure.

#### E. coli and total suspended solids

In river concentrations of E. coli were estimated using United States Environmental Protection Agency (USEPA) methods 1103.1 [36] and 1604 [37], which represent the accepted industry standard for analysis of *E*. *coli* in aquatic systems. Briefly, water grab samples were collected at transect points and vacuum-filtrated through sterile gridded nitrocellulose membrane filters (0.45 μm pore size; Thermo Fisher Scientific, Waltham, Massachusetts, USA). Following filtration of each sample, the sides of the funnel were rinsed twice with 20–30 mL of sterile reagent-grade de-ionized (DI) water. Filters were then aseptically transferred to the surface of a RAPID’E.coli2 (BIORAD, Hercules, California, USA) agar plates, and incubated at 37°C for 24 hours prior to colony enumeration.

Although limitations exist to the use of *E*. *coli* for predicting the presence of other waterborne pathogens [25, 38] numerous studies have demonstrated the efficacy of its use as an indicator organism when evaluating levels of fecal contamination of water sources and potential for transmission of microorganisms between humans and wildlife [39–43]. The results of both field and laboratory studies also indicate that enteric bacteria are commonly associated with suspended solids in aquatic environments, and this association may influence microbial transport dynamics [44, 45].

Total suspended solids (TSS, mg/L) were were evaluated using the USEPA method 160.2 [46]. Briefly, water samples were filtered (Millipore AP-40; Thermo Fisher) and dried at 103–105°C for one hour before being weighed. This drying cycle was repeated until a constant weight was obtained.

### Wildlife biomass and fecal count data

Wildlife biomass estimates were derived from aerial wildlife survey data collected by the Botswana Department of Wildlife and National Parks (DWNP) during the 2012 dry season, and are represented as Large Stock Units (LSU)/km^2^ [47, 48]. DWNP aerial census data was collected using a stratified systematic transect sampling design and data generated from the surveys were analyzed using Jolly’s (1969) method for sampling blocks of unequal size to obtain wildlife biomass estimates [49]. Fecal count (FC) data were collected along points using a line transect survey method as previously described by Jobbins and Alexander (2015) [50]. Briefly, we established 55 fecal transects, odd number transects were also used for water sampling as discussed above. Each transect was 50m in length and spaced 500m apart perpendicular to the Chobe River starting at the confluence of the Chobe and Zambezi Rivers. Dry season FC data (n = 1565) were collected in July, August, and September, 2012. Fecal deposits were identified at one meter intervals along and perpendicular to the transect as far as the observer could see. Each fecal samples was evaluated by an experienced Basarwa tracker who identified species and approximate age of the sample. Elephant FC data (n = 551) were analyzed separately from other wildlife species (n = 1014) in order to assess the potential influence of this large herbivore on system-level seasonal patterns of *E*. *coli* concentrations in the Chobe River.

### Data Analysis

#### Simultaneous autoregressive (SAR) spatial models

All statistical analyses were conducted using the open source integrated programming environment R (R Core Team, 2013) unless otherwise noted. Simultaneous autoregressive (SAR) spatial models were used to analyze associations between water quality variables and spatial and environmental factors. An assumption of the SAR model is that the response at each location is a function of both the explanatory variable at that location, as well as the values of the response at neighboring locations [51, 52]. This approach takes into consideration spatial autocorrelation structure present in the data. Spatial models were run using the {spdep} [53] and {ncf} [54] statistical packages following methodology described by Kissling and Carl (2008, [52]). Equations for the SAR model include the standard terms for predictors (***Xβ***) and errors (***ε***) used in ordinary least squares (OLS) regression, as well as a row standardized spatial weights matrix (**W**) which describes the relationships between neighboring observations. The matrix **W** is not required to be symmetrical and allows for inclusion of anisotropy (i.e. directionality). SAR models can take three different forms depending on whether the spatial autoregressive process occurs only in the error term (SARerr), only in the response variable (SARlag), or in both the response and predictor variables (SARmix) [55–57]. The SARerr equation takes the form

Y= Xβ+ λWu+ ε

where ***λ*** is the spatial autoregression coefficient and ***u*** is the spatially-dependent error term. In the SARlag equation

Y= ρWY+Xβ+ ε

*ρ* is the autoregression parameter which, combined with ***W***, describes spatial autocorrelation in the response variable ***Y***. The SARmix model

Y= ρWY+Xβ+ WXγ+ε

includes the term (***WXγ***), which describes the regression coefficient (***γ***) of the spatially lagged predictor variables (***WX***). For a more details on the formulation of SAR equations and the estimation of the covariance matrices see Anselin (2002, [57]), Haining (2003, [55]), and Fortin and Dale (2005, [58]).

We classified floodplain as being present from transect point 55 in the Park to point 25 in Town, immediately upstream of where the Chobe River crosses the Mambova fault (Fig 1). Floodplain was considered absent from the eastern edge of the fault line to the Chobe River’s confluence with the Zambezi River (transect points 23–1). Separate wet and dry season models were fit for water quality, land use, floodplain, and fecal count covariates. Due to the shorter collection period for abiotic data, separate models were analyzed for 2012–2013 (Model 1) and 2011–2014 (Model 2). The influence of seasonal fecal loading on wet and dry season *E*. *coli* concentrations was also explored (Model 3). Elephant-specific and other wildlife species fecal counts were analyzed to assess the potential influence of terrestrial fecal loading on dry season *E*. *coli* concentrations. Data from even-numbered transects were combined with values from the nearest odd-numbered downstream transect point for analysis in order to scale up FC data collected every 500m to our water sampling transects (every 1km). In addition to assessing within-season associations, we also evaluated the influence of dry season fecal loading on mean *E*. *coli* concentrations during the first three months of the wet season (December-January). The models were initially fit using ordinary least squares (OLS) regression and Lagrange multiplier (LM) diagnostics were calculated from model residuals in order to identify the nature of spatial dependence (i.e. error, lag, mixed). LM test statistics allow for distinction and selection between the performance and appropriateness of competing spatial models [59]. A natural log and Box-Cox transformations were applied to seasonal water quality and fecal count variables, respectively, to better approximate a normal distribution prior to fitting regression models.

#### Geostatistical analysis

Spatial autocorrelation (SAC) patterns in wet and dry season *E*. *coli* and TSS data were examined using the Moran’s Index tool in ESRI ArcGIS (Ver. 10.2, Redlands, California). Ordinary kriging in ArcMap Geostatistical Analyst Extension was used to interpolate and map spatial patterns of *E*. *coli* and TSS concentrations. Ordinary kriging is essentially a generalized linear regression technique that utilizes the underlying spatial correlation structure of data to derive optimal weights in order to estimate values at un-sampled points. As a best linear unbiased estimator, ordinary kriging also aims to minimize the variance of model errors. In order to satisfy model assumptions that data are approximately normally distributed, we applied a log transformation to *E*. *coli* and TSS data prior to model fitting, and verified univariate normality using normal qqplots. Eleven theoretical semiovariogram models (Circular, Spherical, Tetraspherical, Pentaspherical, Exponential, Gaussian, Rational Quadratic, Hole effect, K-Bessel, J-Bessel, Stable) were fitted through interactive plotting to explore spatial continuity in wet and dry season *E*. *coli* and TSS data. The influence of anisotropy was also investigated, and model prediction performance was evaluated by cross-validation. Model suitability was assessed based upon the criteria that root-mean-square error (RMSE) and average standard error are minimized, the standardized mean error was close to zero, and the root-mean square standardized error was close to one. A smaller ratio between root-mean-square prediction errors from cross-validation also provides an indication that prediction standard errors are within reasonable limits (ESRI 2001).

#### Time series decomposition

Temporal patterns of *E*. *coli* and TSS were analyzed by seasonal and trend decomposition using local regression (STL). STL is an iterative nonparametric graphical method for describing a time series by its additive seasonal, trend (i.e. a long-term increase or decrease in the data), and remainder (residual) components of variation using locally-weighted regression (LOESS) smoothing [60]. STL proceeds through a series of two nested recursive loops whereby a weight is defined for each observation. Large residuals (e.g. outliers) are assigned smaller weights and small residuals, larger weights, which has the effect of minimizing or smoothing the influence of outliers, resulting in a robust procedure for visualizing the nonlinear patterns and periodic components of variability within a time series [60].

## Results

### Spatiotemporal water quality patterns

**Fig 3:**
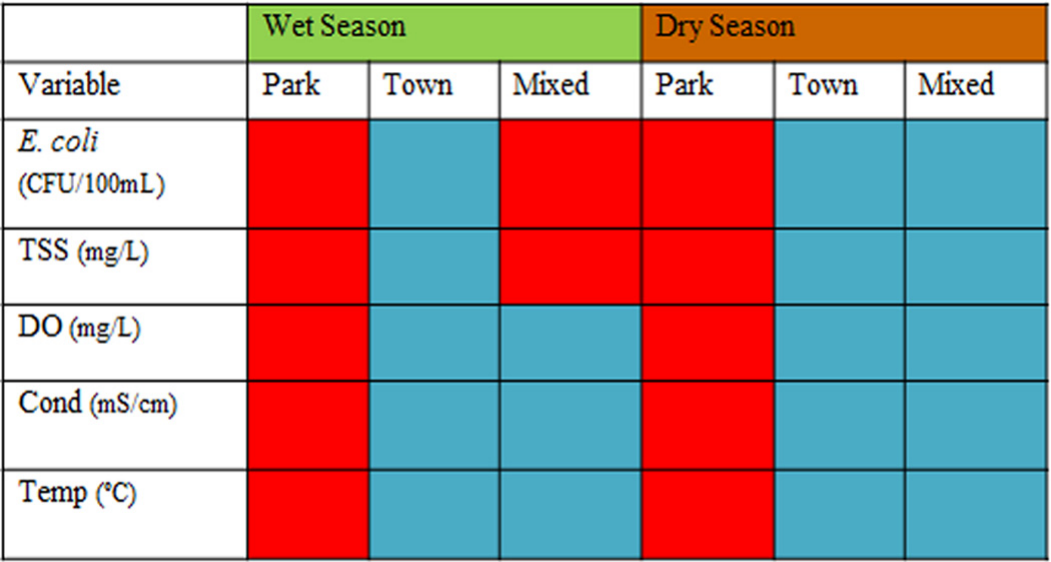
Seasonal relationships among water quality variables are summarized for the different land use classes. Red cells indicate that the mean value for each variable and land use is greater than the global mean for the study area, while blue symbolizes values below the global mean.

**Fig 4:**
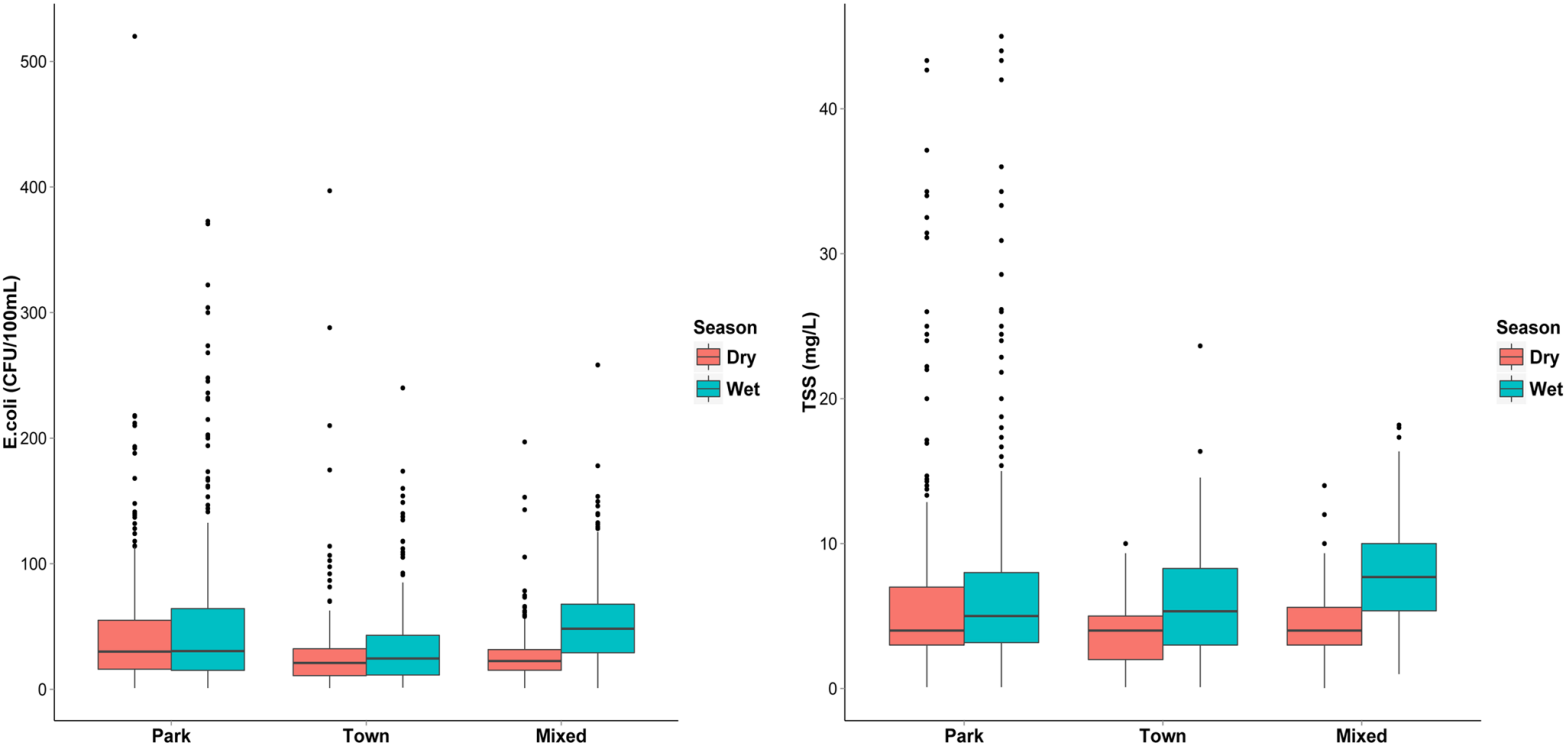
Box plots with outliers retained showing seasonal patterns of *E*. *coli* (CFU/100mL) and TSS (mg/L), in the Chobe River by land use class. Variability in the concentration data was high across all land use classes during both seasons, but was greatest within the Park and generally lower in Town and Mixed land use. Median wet season *E*. *coli* and TSS were similar to dry season median concentrations in Park and Town landuse. In Mixed land use, median wet season concentrations were more than twice those observed during the dry season.

**Fig 5:**
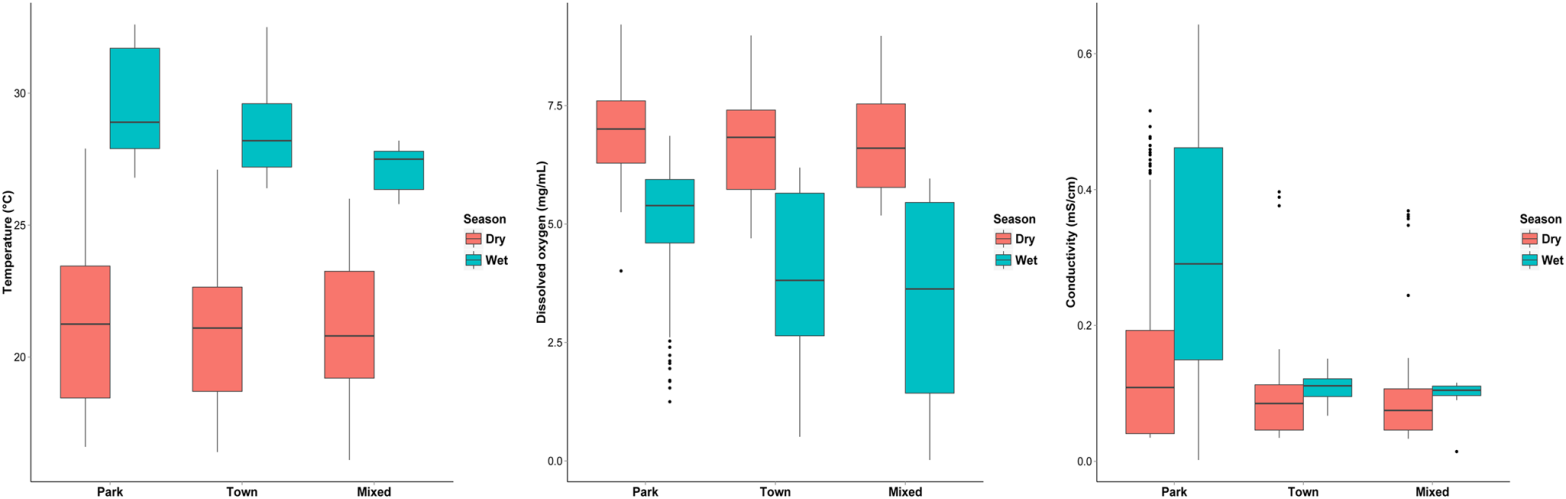
Box plots with outliers retained showing seasonal patterns of water temperature (°C), dissolved oxygen (mg/L), and specific conductivity (mS/cm) in the Chobe River by land use class. Median values were similar across all three land use classes during the dry season, but differed substantially during the wet season. Median wet season DO was highest in the Park (despite having higher water temperature) compared to downstream levels, which may reflect greater primary productivity from vegetation in the Chobe River floodplain and its associated wetland systems. Median seasonal conductivity and interquartile range were highest in Park land use, with monthly median values ranging from a low of 0.04 mS/cm (May) at peak flood height, to a high of 0.42 mS/cm (October) when water levels were lowest. The range of observed seasonal values for water temperature, DO, and conductivity for the Chobe River were comparable to those recorded by Mackay et al. (2011) for the Okavango River Delta in 2006–2007 [61].

Seasonal water quality statistics for the three land use classes are shown in Table 1, and seasonal relationships among water quality variables are summarized for the different land uses classes in Fig 3. Box plots show overall seasonal patterns of *E*. *coli* and TSS (Fig 4), and abiotic water quality variables (Fig 5) with outliers retained. Dry season mean *E*. *coli* in Park land use (42 CFU/100mL) was 37% higher than in Town and 43% higher than in Mixed land use. Maximum *E*. *coli* in the Park (519 CFU/100mL) was more than twice that observed in Town and Mixed land use.

**Table 1:** Arithmetic mean, maximum, and standard deviation of seasonal *E*. *coli* and TSS concentrations by land use class, calculated from water quality samples collected bimonthly from July 2011- March 2014.

	E.coli (CFU/100mL)						TSS (mg/L)					
	Wet			Dry			Wet			Dry		
Location	Mean	Max	sd	Mean	Max	sd	Mean	Max	sd	Mean	Max	sd
Mixed	56	257	38	27	197	21	7.9	18.2	3.5	4.3	14.0	2.3
Town	36	397	39	29	239	40	6.0	23.6	3.8	3.9	10.0	2.2
Park	54	373	63	42	519	44	7.3	45.0	7.3	6.4	43.3	6.7

**Fig 6:**
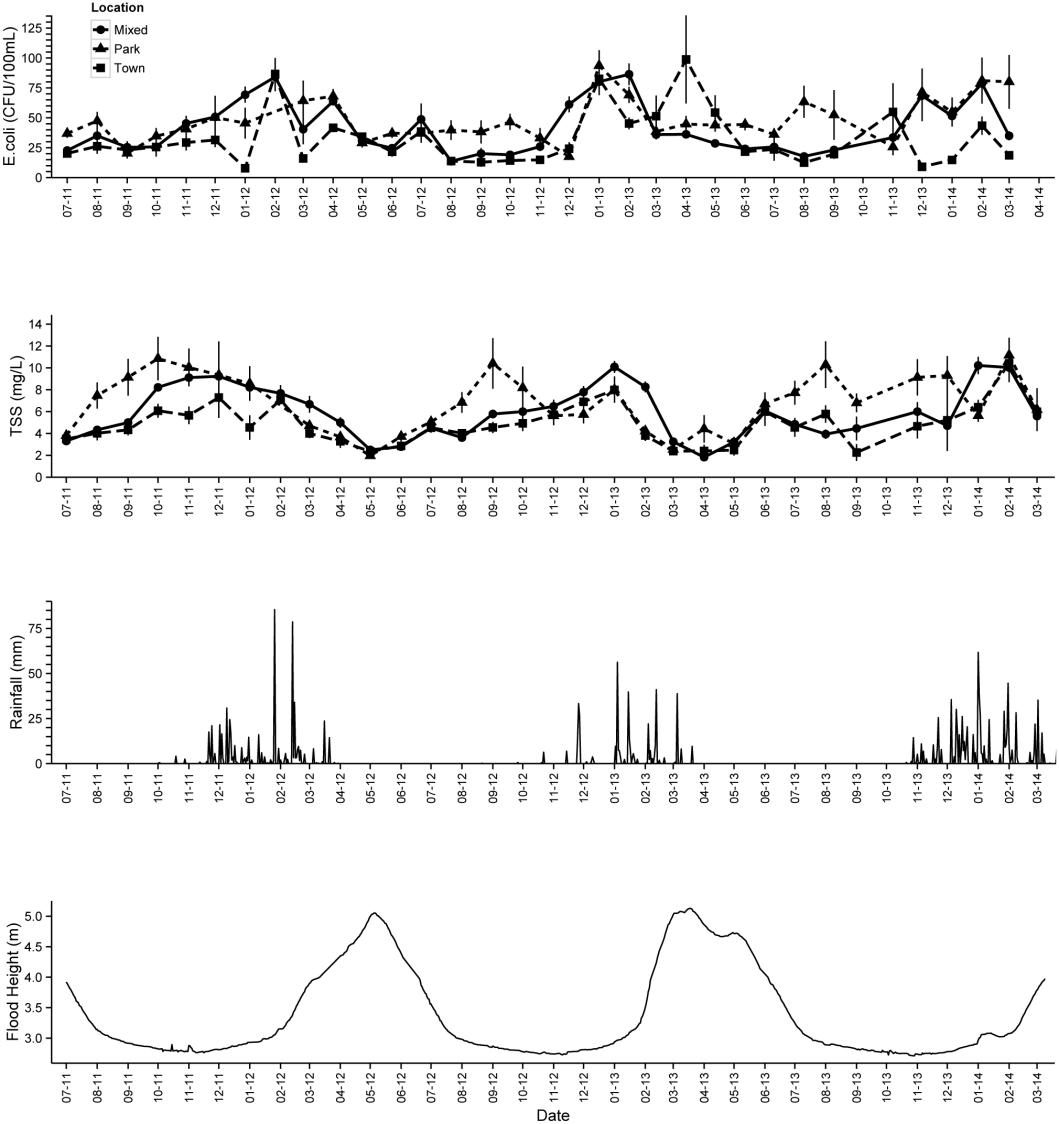
Monthly time series of bi-monthly water sampling data showing temporal and spatial variation of mean *E*. *coli* (CFU/100mL) and TSS (mg/L) concentrations with standard errors by month and land use class in relation to daily precipitation (mm), and Chobe River height (m).

**Fig 7:**
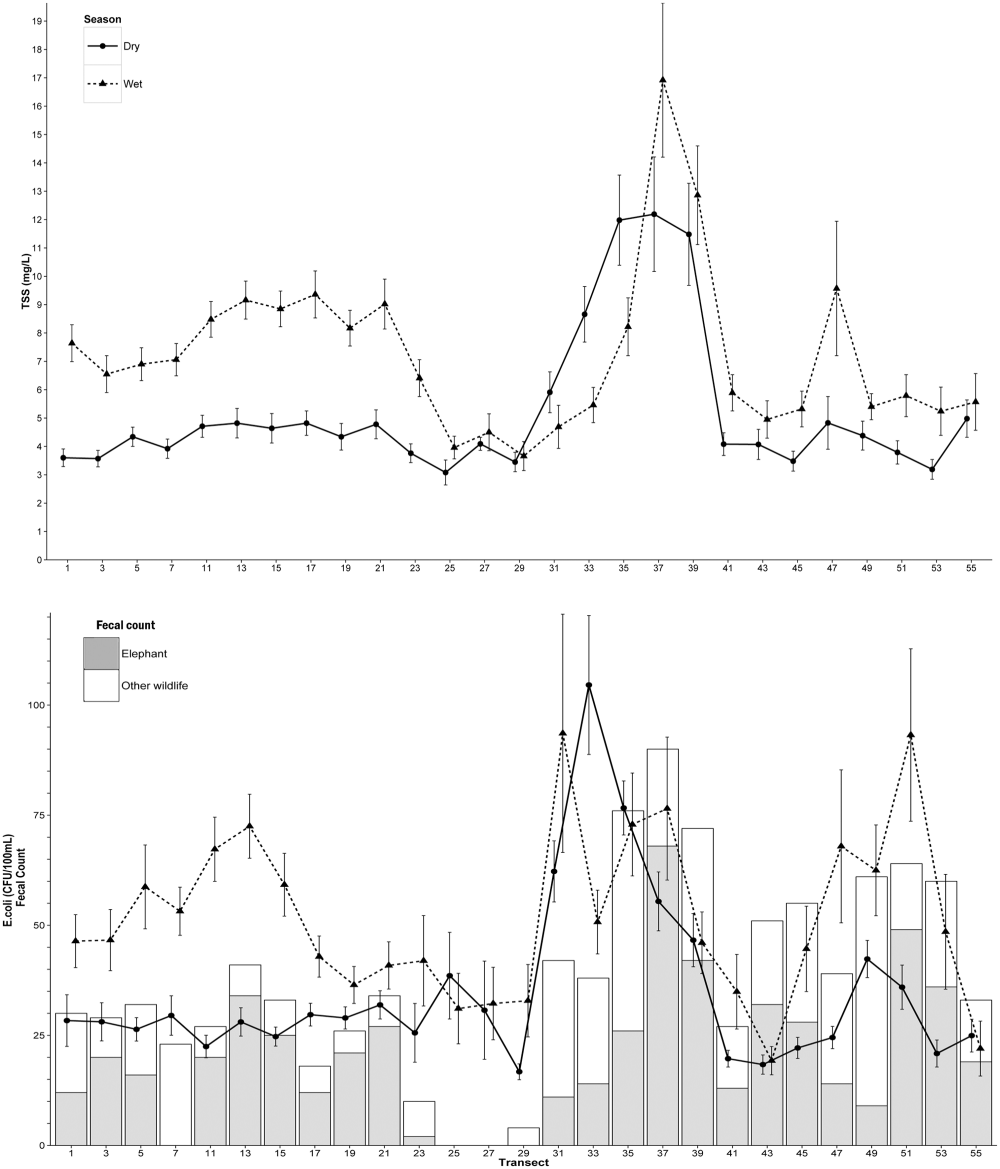
Arithmetic means of bi-monthly water sampling data by transect point showing seasonal and spatial variability of *E*. *coli* (CFU/100mL) and TSS (mg/L) concentrations with standard errors. Transect points were assigned to three general land use types and proceed in an upstream direction from Mixed-use (1–17), Town (19–29), Park (31–55).

A strong seasonal pattern is evident in both *E*. *coli* and TSS time series, with mean *E*. *coli* concentrations increasing in late November coinciding with the onset of heavy wet season rainfall, and then declining at the beginning of the dry season in Mixed and Town land use (Fig 6). In contrast, TSS concentrations were highest in the late dry season and lowest in May when flood waters peaked. Spatial plots of average seasonal *E*. *coli* and TSS concentrations (Fig 7) show points 31–39 were influential in driving high dry season levels observed in Park land use. Wet season mean *E*. *coli* concentrations in the Park were more than twice as high compared to points in Town. Values also varied widely even over short distances. For example, mean dry season *E*. *coli* at transect point 33 in the Park was more than six times that of the value recorded at point 29 in Town, just two kilometers downstream.

**Fig 8:**
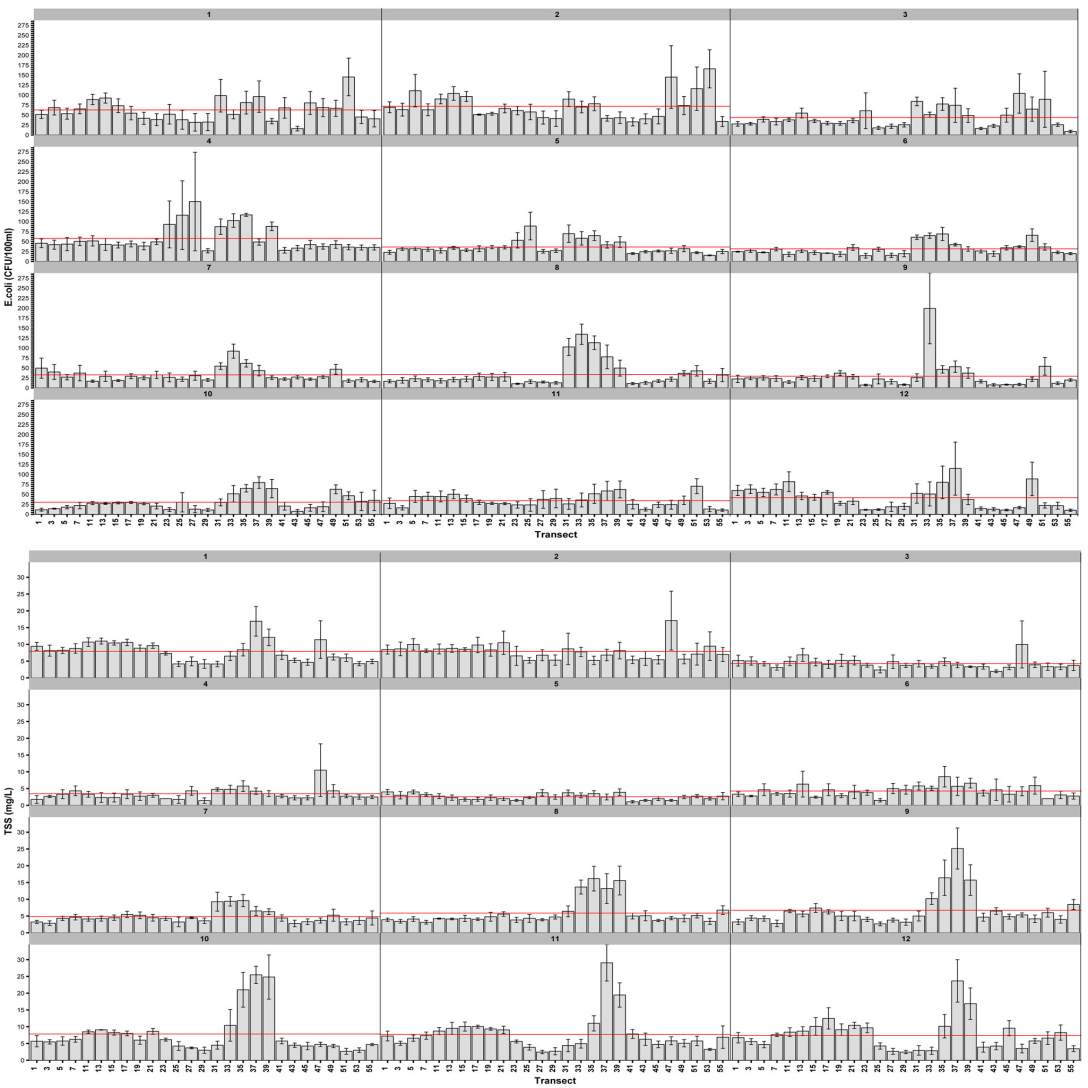
Mean E. coli and TSS time series data by individual month and transect with standard errors. Monthly averages for the study area are indicated by horizontal lines.

Monthly mean values for both *E*. *coli* and TSS concentrations are provided by transect point in Fig 8. *E*. *coli* and TSS concentrations were highest in January and February, before experiencing a decline in March. Interestingly, *E*. *coli* concentrations measured at transect points 23–27 in Town, which were generally lower than in Park and Mixed land use classes during all other months, spiked in April coinciding with peak flood height. In the Park, elevated *E*. *coli* concentrations occurred between July and October, when comparatively low levels were recorded in Mixed and Town land use. Mean TSS concentrations were also highest in the Park between the months of July and December.

### STL time series decomposition

**Fig 9:**
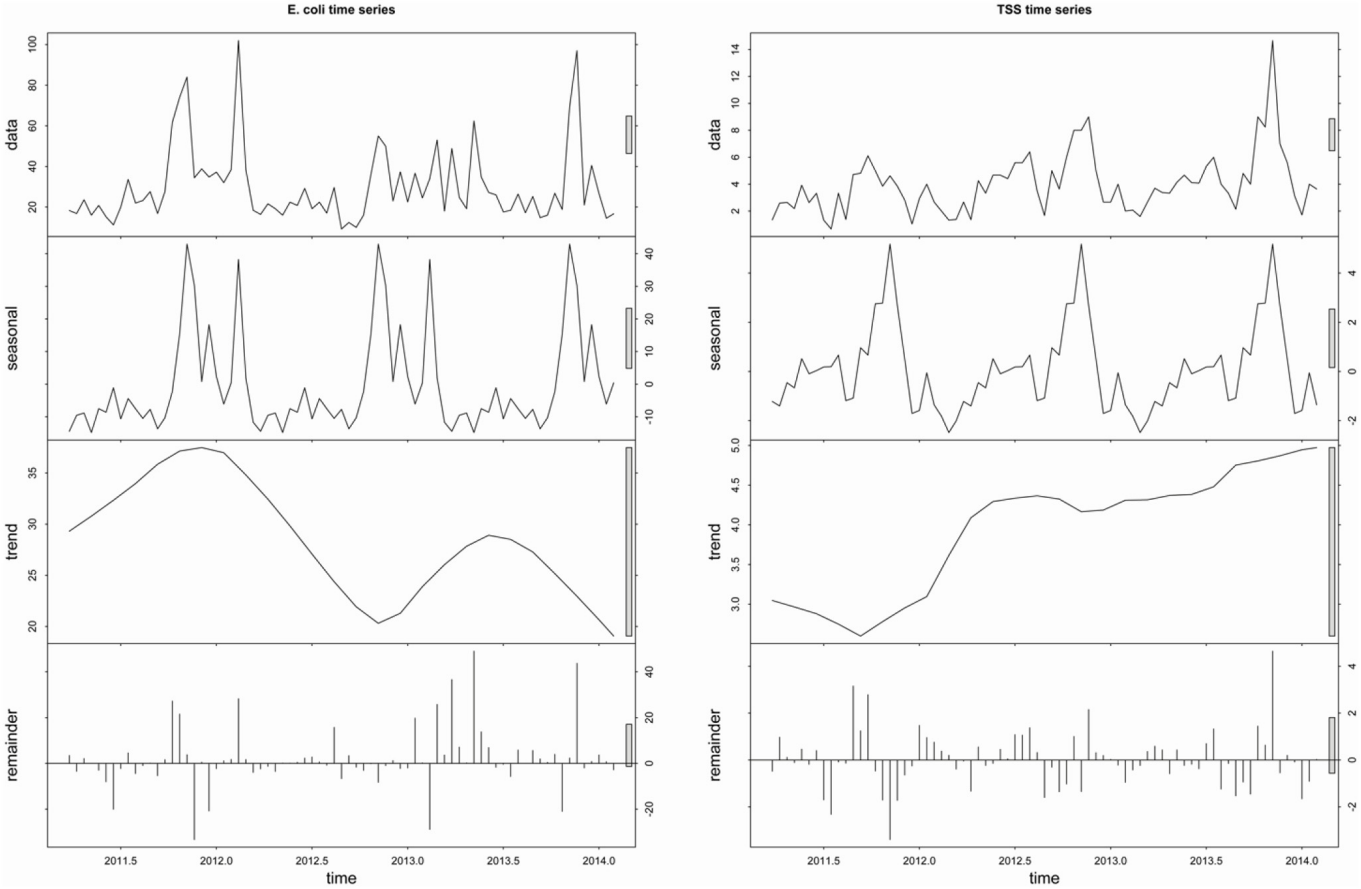
Seasonal and trend decomposition using loess (STL) for *E*. *coli*. and TSS water quality data collected bi-monthly from July 7, 2011 –March 19, 2014. Raw data are displayed in the top panel as averaged values for all transect points with a two week sampling frequency, followed by seasonal, trend, and residual components. Scale differs for each of the components so relative magnitude is indicated by the gray bars on the right side of the panels. The bar in the top panel represents a single unit of variation, with larger bars indicating a smaller amount of variation attributable to a particular component.

The results of time series decompositions of *E*. *coli* and TSS data show the strong influence of seasonal factors on Chobe River water quality dynamics (Fig 9). The lack of an upward or downward trend indicates that concentrations of *E*. *coli* and TSS did not exhibit any detectable directional change over the course of the study period. Substantial variation attributed to the residual component of the time series data suggests factors separate from those operating on seasonal time scales impact water quality in the system. Finer-scale seasonal variations in *E*. *coli* and TSS among the different land use classes were also investigated (S1–S3 Figs). Seasonal contributions to the variability of *E*. *coli* concentrations was highest in the Park and lowest in Town, while the effect of seasonality on TSS variability generally decreased in a downstream direction from Park to Mixed land use classes.

### Kriged water quality maps

**Fig 10:**
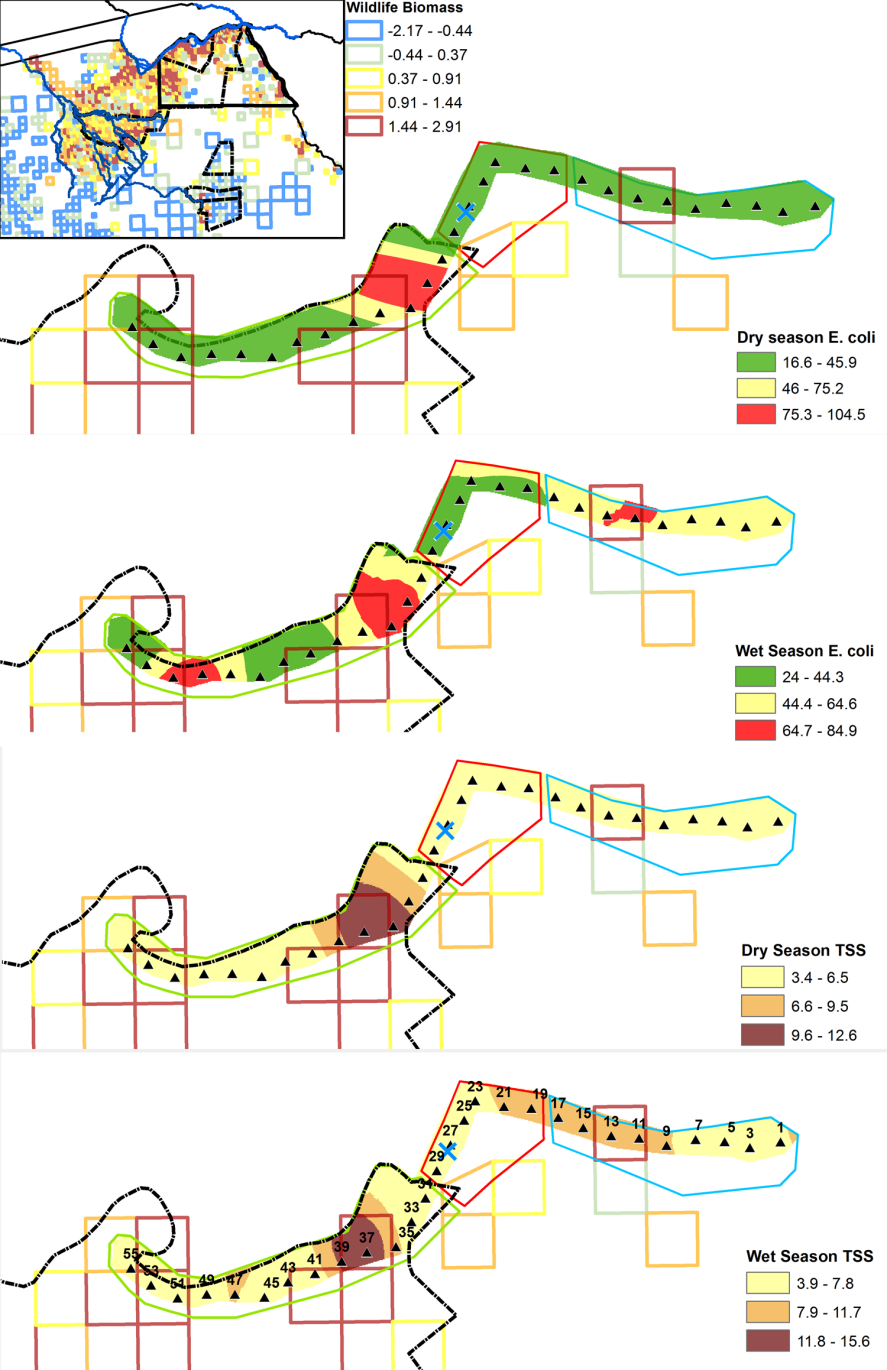
Raster surfaces of seasonal mean *E*. *coli* (CFU/100mL) and TSS (mg/L) estimated using ordinary kriging, overlaid with 2012 dry season aerial survey data showing log wildlife biomass (kg/km^2^). The Chobe National Park (dashed black line), Kasane water plant (light blue cross), transect points (triangles) and general land use classes: Park (green solid line), Town (red), and Mixed (light blue) are also shown. Close spatial overlap between areas of high and low *E*. *coli* and TSS concentrations can be seen during both wet and dry seasons.

*E*. *coli* and TSS exhibited a significant clustered spatial autocorrelation pattern in both wet and dry seasons (S1 Table). Semivariogram analysis of log mean *E*. *coli* and TSS concentrations showed a Gaussian semivariogram model with anisotropy performed best for dry season log *E*. *coli* and TSS according to model cross-validation. Wet season *E*. *coli* semivariance was best described by a J-Bessel model with anisotropy, while cross-validation showed a J-Bessel model without anisotropy performed best in predicting wet season TSS concentrations. Water quality maps (Fig 10) of both *E*. *coli* c and TSS value showed close spatial overlap across seasons, particularly in Park land use.

### Landuse and floodplain influence

Significant spatial error and lag processes (p<0.0001) were present in all OLS models for both wet and dry seasons. SARmix model was used to correct for SAC present in both the response and predictor variables. Residuals from all seasonal models were not significant at a 0.05 α-level using the Breusch-Godfrey LM test for serial correlation, indicating that the SARmix models were effective in accounting for SAC. In Model 1 (Table 2) only TSS was found to be statistically significant and positively associated with log-mean *E*. *coli* (p<0.0001) concentrations during the wet season. Coefficients for the dry season model were significant for dissolved oxygen (p<0.0001), Park land use (p = 0.0009), and presence of floodplain (p = 0.044). Park land use and floodplain presence were both positively correlated with dry season *E*. *coli*, while lower DO levels were associated with higher *E*. *coli* concentrations. In Model 2 (Table 3), TSS was the only wet season variable significantly associated with *E*. *coli* (p<0.0001), while in the dry season the coefficients for Park land use (p = 0.0009) and floodplain presence (p = 0.016) were again significant.

**Table 2:** Model 1 –SAR mixed model results testing the effects of explanatory variables from 2012–2013 on seasonal log-mean *E*. *coli* (CFU/100mL).

	Dry Season				Wet Season			
Variable	Coefficient	Std. error	Z-value	*P*-value	Coefficient	Std. error	Z-value	*P*-value
LN_TSS	0.1477	0.0848	1.7422	0.081	0.7507	0.1512	4.9655	**<0.0001**
LN_Cond	-0.0411	0.0815	-0.5042	0.614	0.0501	0.1425	0.3515	0.725
LN_DO	-2.0939	0.3386	-6.1833	**<0.0001**	0.0330	0.1057	0.3121	0.755
LN_Temp	-0.6993	0.4740	3.1704	0.056	-1.0189	1.3822	-0.7372	0.461
Land use (reference is Mixed)								
Park	1.5029	0.3359	0.4456	**0.002**	-0.1213	0.7975	-0.1521	0.879
Town	0.1497	0.3401	0.3585	0.656	-0.5214	0.6105	-0.8540	0.393
Floodplain (reference is Absent)								
FldPln Present	0.6576	0.3270	2.0108	**0.044**	-0.3173	0.5970	-0.5316	0.595

**Table 3:** Model 2 –SAR mixed model results testing the effects of explanatory variables from 2011–2014 on seasonal log-mean *E*. *coli* (CFU/100mL).

	Dry Season				Wet Season			
Variable	Coefficient	Std. error	Z-value	*P*-value	Coefficient	Std. error	Z-value	*P*-value
LN_TSS	0.0316	0.0361	0.8758	0.381	0.3306	0.0502	6.5885	**<0.0001**
Land use (reference is Mixed)								
Park	1.0802	0.3270	3.3034	**0.0009**	0.6327	0.4028	1.5708	0.116
Town	-0.0992	0.2495	-0.3976	0.691	-0.0694	0.3096	-0.2243	0.823
Floodplain (reference is Absent)								
FldPln Present	0.5470	0.2265	2.5151	**0.016**	0.2477	0.2215	1.1179	0.264

### Wildlife influence

Analysis of the relationship between terrestrial fecal loading and bacterial concentrations showed a significant positive influence of elephant-specific fecal count (FC) (p = 0.017) and other wildlife species FC (p = 0.001) on *E*. *coli* concentrations in the dry season (Table 4). Mean dry season elephant FC (p = 0.029) and other wildlife FC (p = 0.006) were also significant predictors of *E*. *coli* concentrations during the first three months of the wet season (December-February). Areas of high log wildlife biomass (LSU/km^2^) estimated from 2012 dry season showed close spatial overlap between aerial wildlife survey data with locations of high mean *E*. *coli* and TSS concentrations in the kriged water quality maps, particularly during the wet season (Fig 10). Graphical comparisons of FC in relation to mean seasonal *E*. *coli* and TSS by transect (Fig 6) showed transect locations with high dry season terrestrial fecal deposition were associated with higher mean concentrations of bacteria and suspended solids during both wet and dry seasons. This relationship was most apparent at points adjacent to floodplain habitat.

**Table 4:** Model 3—SAR mixed model results testing the influence of terrestrial within and between-season fecal loading on log-mean *E*. *coli* (CFU/100mL).

	Dry Season				Wet Season			
Variable	Coefficient	Std. error	Z-value	*P*-value	Coefficient	Std. error	Z-value	*P*-value
Ele_FC^+^	0.1324	0.0552	2.4001	**0.017**	-	-	-	-
Wild_FC*	0.2201	0.0669	3.2883	**0.001**	-	-	-	-
Dry_ele_FC^+^	-	-	-	-	0.1297	0.0593	2.1863	**0.029**
Dry_wild_FC*	-	-	-	-	0.2233	0.0816	2.7364	**0.006**

Dry season fecal counts for elephant+ and for all other wildlife species excluding elephants*, were compared to dry season *E. coli* concentrations.

We also assessed the influence of dry season fecal count data on *E. coli* concentrations at the start of the wet season (December-February).

## Discussion

In this dryland river system, we observed important water quality declines during the wet season across all land use classes associated with seasonal rainfall and flood pulse dynamics. Patterns of TSS were predictive of *E*. *coli* concentrations during the wet season. *E*. *coli* concentrations at the start of the wet season were also significantly associated with dry season fecal loading by elephant and other wildlife with, suggesting storm water and sediment runoff are major factors influencing wet season bacterial loads. Overall water quality trends were consistent with the dry season distribution of wildlife in this system where the densities occurred in the Park and lower densities occurred in urbanizing areas. Dry season concentrations of *E*. *coli* were significantly higher at locations with extensive floodplain habitat and showed close spatial agreement with estimates of wildlife biomass derived from aerial survey data.

We observed the greatest declines and variability in water quality in Park land use over both wet and dry seasons. Water quality in Mixed land use was less variable than in the Park and only demonstrated declines in the wet season, while Town land use had generally better water quality over both seasons (Figs 5 and 6). Dry season water quality declines in the Park were especially notable given the considerably lower average concentrations of *E*. *coli* and TSS recorded in the other land use classes over the same period. Water quality dynamics in Mixed and Town land use were typical of those observed in other flood-pulse systems in which water quality declines frequently coincide with the rising limb of flood events [62]. Dry season water quality declines observed in the Park, however, were notably decoupled from precipitation and flood-pulse events. According to research by Alexander et. al [9], recurrent diarrheal disease outbreaks in the Chobe District between 2006–2009 and 2011–2012 also coincided with the timing of flood and rainfall events in the system. A first outbreak period was identified in the wet season from January through March, while the second lasted from July to October coinciding with flood recession and seasonally low water levels. The results of this and other studies, suggest that a one health approach, which importantly incorporates consideration of the environment, is necessary to understanding the complex dynamics that link humans, animals, and the environment to resultant health outcomes.

Surface water quality declines are typically associated with human habitation and landscape change such as agriculture, livestock husbandry, industry, and urban development [63, 64]. But our results suggest that these expectations may not hold true in areas where wildlife populations concentrate at very high densities along perennial dryland systems such as the Chobe River. Activities of elephant and other large animals play an essential role in maintaining the long-term integrity of river corridors in southern Africa, adding nutrients and increasing patch heterogeneity of the riparian landscape [65]. In areas where wildlife concentrate in riparian corridors, however, this influence may extend beyond the terrestrial environment to impact seasonal water quality dynamics.

Arid and semi-arid regions occupy a large proportion of the Earth’s total land surface but perennial rivers occupy only a small fraction of these landscapes [66]. As the only permanent surface water source in the 21,000 km^2^ district, the Chobe River is major influence on the ecology, movement, and life history of the region’s wildlife [7, 67, 68]. Chobe National Park has an area of 11,700 km^2^, of which the Chobe River spans ca. 60 km or one half of one percent of the CNP’s total land area. Development of artificial waterholes a management strategy within the CNP has generally been avoided as these man-made features can potentially transform patterns of wildlife distributions and landscape use, even when natural surface water sources are available [69].

Wildlife access to the Chobe River outside of the CNP’s boundaries is becoming increasingly restricted due to rapid expansion of urban land use within the towns of Kasane and Kazungula [30]. The lack of reliable alternative surface water sources in the CNP during the dry season constrains water-dependent wildlife species to forage within relatively short distances from the river [70]. While many species of browsers and grazers utilize the Chobe River and floodplain during the dry season, our data show that elephants contributed disproportionately to fecal loading within the riparian corridor. Estimated average dry season elephant density within the Chobe National Park based on aerial survey data was seven LSU/km^2^, but this increased to 13 LSU/km^2^ within a five kilometer buffer distance from the Chobe River. The high concentration of large animals along the Chobe riverfront, while a boon for the region’s growing wildlife safari industry, may significantly impact both vegetation and soil communities, in addition to water quality.

While seasonal influences on surface water quality have been described in other African river systems [71–74], this study is among first to identify large, free-ranging wildlife populations as having a significant influence on spatiotemporal patterns of water quality. Although recent research has led to an improved understanding of how large animals can function as ecosystem engineers impacting the availability of resources to other species [65, 75], there has been little recognition of the potential influence free-ranging wildlife on aquatic systems. Management of floodplains and riparian habitat seldom involves consideration of terrestrial wildlife effects on river system integrity. Our results highlight the importance of maintaining large, contiguous areas for animals to access surface water in order to minimize the potential impacts of overcrowding on riparian habitat and water quality in vulnerable dryland systems.

## Conclusion

Seasonal patterns of rainfall and hydrology often control the distribution of water-dependent wildlife species in dryland regions with limited perennial surface water resources. While it is accepted that livestock, agriculture, and other types of anthropogenic land use can play an important role in water quality declines, wildlife in high densities may also alter the land-aquatic interface in significant ways, potentially influencing water quality dynamics through fecal loading and increased soil erosion. Restriction of water access and compression of wildlife into smaller natural areas could potentially intensify the severity of water quality declines, in addition to increasing existing levels of human-wildlife conflict over available space. Land concessions for competing needs that reduce protected land areas and divert to human development projects may impact water quality dynamics, feeding back to affect the security and welfare of humans and wildlife alike. Future land use and development planning in the Chobe District and other dryland regions of southern Africa should consider potential impacts of landscape alteration on wildlife movements and access to perennial sources of surface water. Efforts to designate and maintain suitable wildlife corridors should be prioritized, both in rural and urban areas. Availability of and access to surface water and floodplain habitat should also be specifically considered in the design and management of protected areas in dryland regions with large, free-ranging wildlife populations.

In southern Africa, surface water resources are under increasing pressure to accommodate competing needs of both humans and wildlife. Challenges associated with managing both surface water quality and protected habitat are expected to escalate during the coming decades, in many cases displacing land areas designated for wildlife. Typically, these conservation land areas are seen as a tool to preserve natural systems and the species that occur there, a purpose that is difficult to rationalize when human needs escalate. However, this work suggests that maintaining sufficient access for water-dependent wildlife along riparian areas in dryland regions may not only be part of a sustainable conservation strategy, but may also be important for securing clean water and improved human health.

## Supporting Information

S1 Fig Seasonal and trend decomposition using loess (STL) of *E*. *coli*. and TSS water quality data collected over the period July 7, 2011 –March 19, 2014 in Park land use. Raw data are displayed in the top panel as averaged values for all transect points with a two week sampling frequency, followed by seasonal, trend, and residual components. Scale differs for each of the decomposed components so relative magnitude is indicated by the gray bars on the right side of the panels. The bar in the top panel can be considered as a single unit of variation, with larger bars indicating a smaller amount of variation attributable to a particular component.
(TIF)

S2 Fig Seasonal and trend decomposition using loess (STL) of *E*. *coli*. and TSS water quality data collected over the period July 7, 2011 –March 19, 2014 in Town land use. Raw data are displayed in the top panel as averaged values for all transect points with a two week sampling frequency, followed by seasonal, trend, and residual components. Scale differs for each of the decomposed components so relative magnitude is indicated by the gray bars on the right side of the panels. The bar in the top panel can be considered as a single unit of variation, with larger bars indicating a smaller amount of variation attributable to a particular component.
(TIF)

S3 Fig Seasonal and trend decomposition using loess (STL) of *E*. *coli*. and TSS water quality data collected over the period July 7, 2011 –March 19, 2014 in Mixed land use. Raw data are displayed in the top panel as averaged values for all transect points with a two week sampling frequency, followed by seasonal, trend, and residual components. Scale differs for each of the decomposed components so relative magnitude is indicated by the gray bars on the right side of the panels. The bar in the top panel can be considered as a single unit of variation, with larger bars indicating a smaller amount of variation attributable to a particular component.
(TIF)

S1 Table Moran’s I test results for spatial autocorrelation in seasonal *E*. *coli* and TSS data. (DOCX)
