# Supplementary figures and images for: Spatiotemporal Variation and the Role of Wildlife in Seasonal Water Quality Declines in the Chobe River, Botswana
Source: PLoS One. 2015 Oct 13;10(10):e0139936. doi: 10.1371/journal.pone.0139936 (PMC4603952; doi:10.1371/journal.pone.0139936)

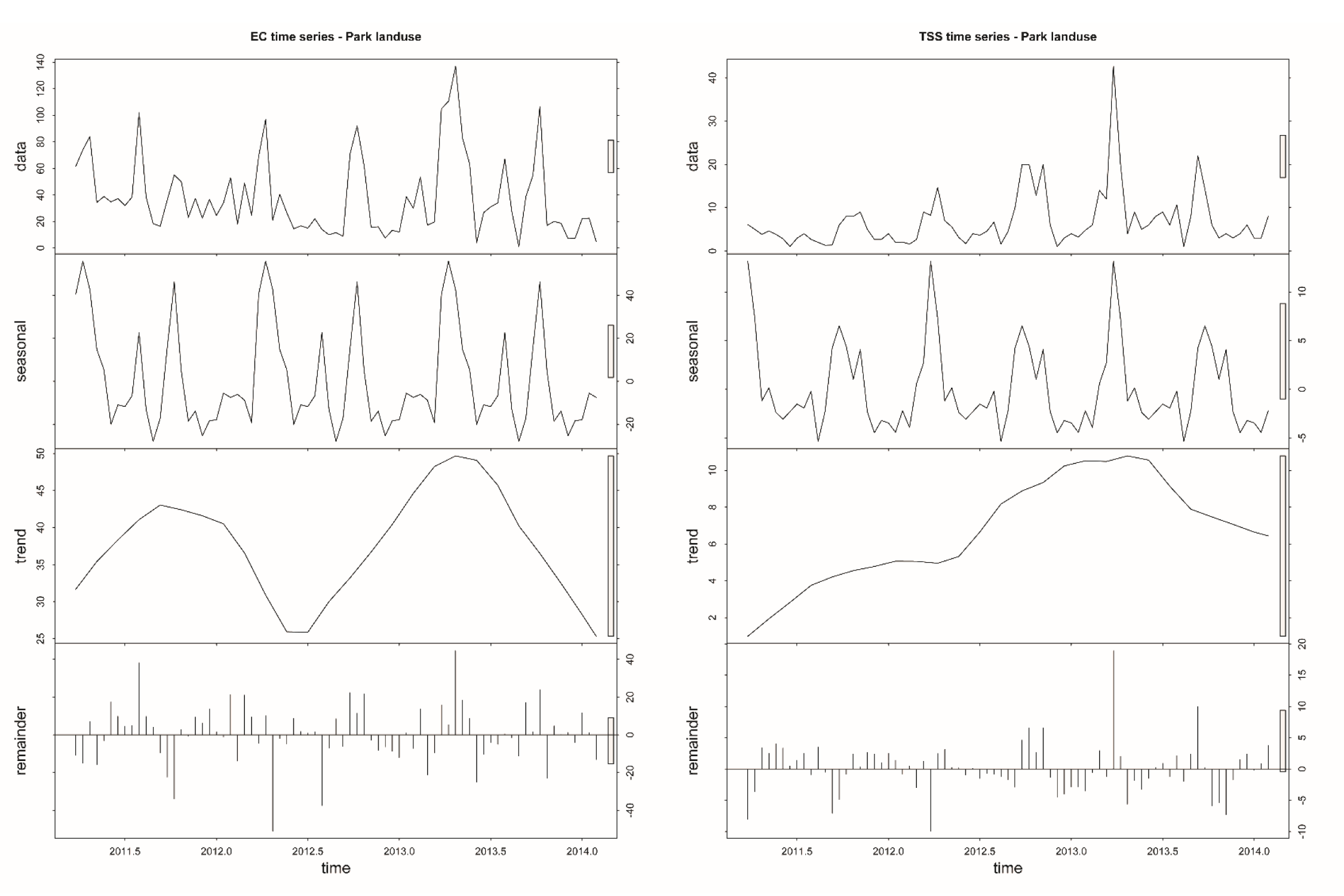

Supplement: S1 Fig — Raw data are displayed in the top panel as averaged values for all transect points with a two week sampling frequency, followed by seasonal, trend, and residual components. Scale differs for each of the decomposed components so relative magnitude is indicated by the gray bars on the right side of the panels. The bar in the top panel can be considered as a single unit of variation, with larger bars indicating a smaller amount of variation attributable to a particular component. (TIF) [file pone.0139936.s001.tif]

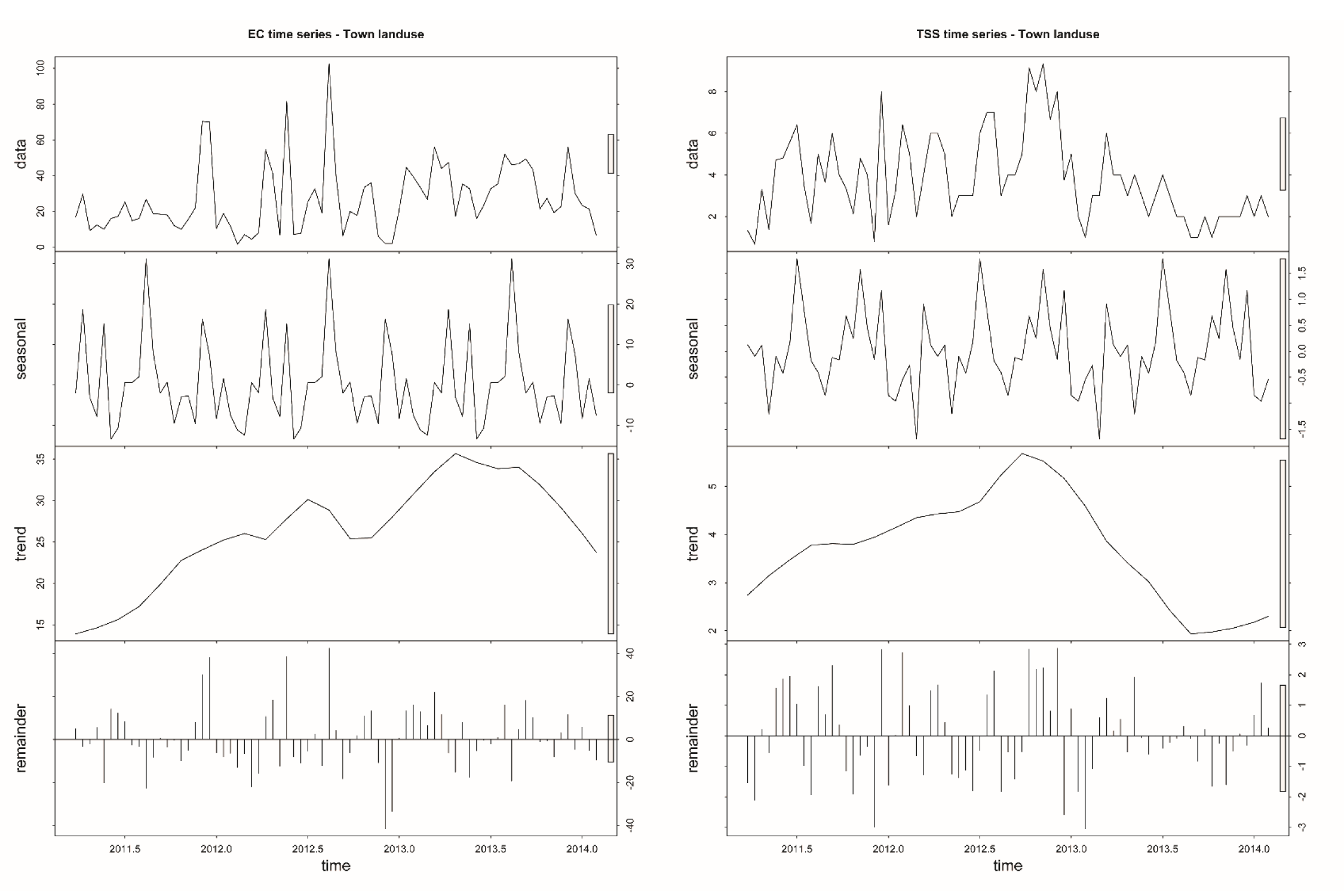

Supplement: S2 Fig — Raw data are displayed in the top panel as averaged values for all transect points with a two week sampling frequency, followed by seasonal, trend, and residual components. Scale differs for each of the decomposed components so relative magnitude is indicated by the gray bars on the right side of the panels. The bar in the top panel can be considered as a single unit of variation, with larger bars indicating a smaller amount of variation attributable to a particular component. (TIF) [file pone.0139936.s002.tif]

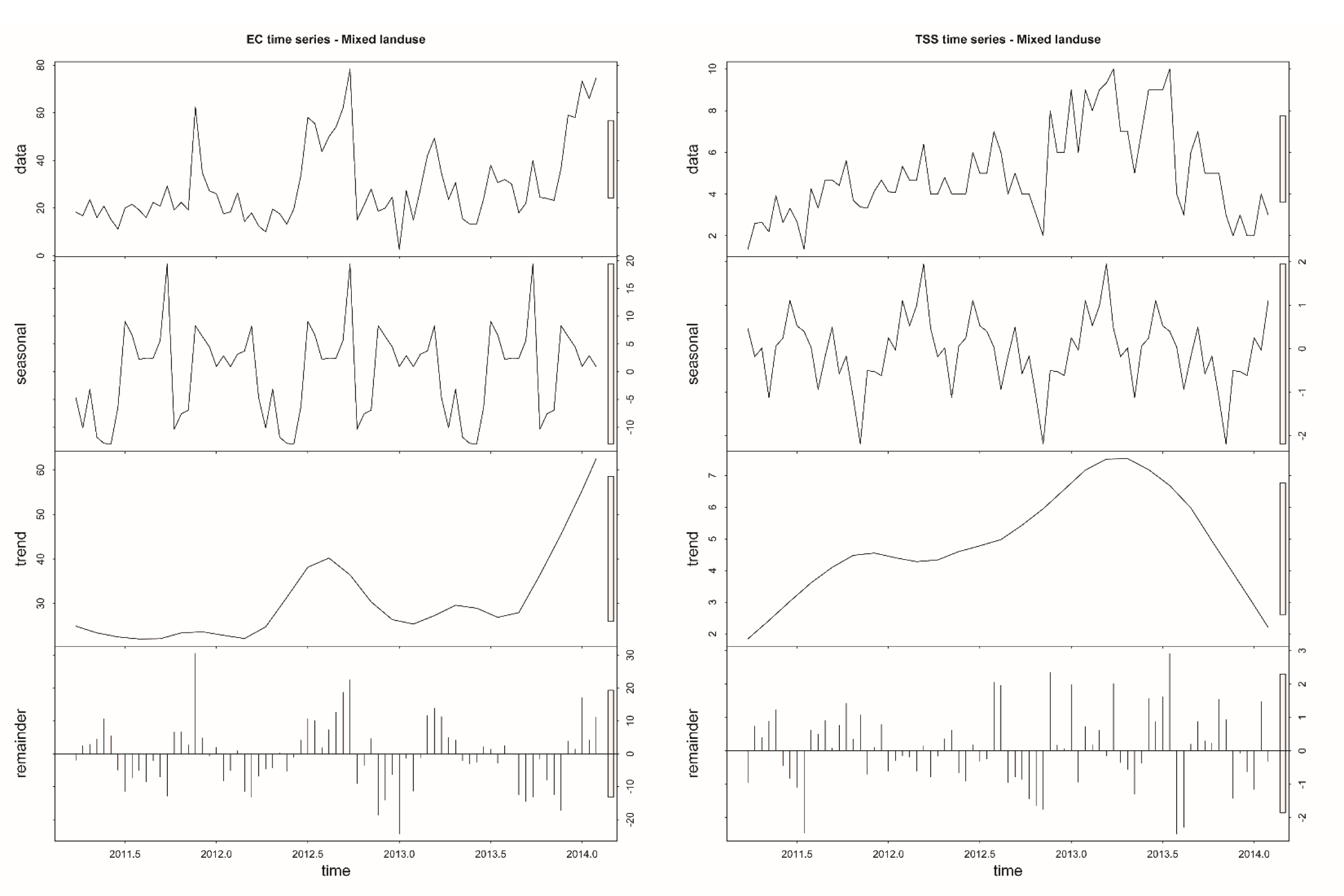

Supplement: S3 Fig — Raw data are displayed in the top panel as averaged values for all transect points with a two week sampling frequency, followed by seasonal, trend, and residual components. Scale differs for each of the decomposed components so relative magnitude is indicated by the gray bars on the right side of the panels. The bar in the top panel can be considered as a single unit of variation, with larger bars indicating a smaller amount of variation attributable to a particular component. (TIF) [file pone.0139936.s003.tif]
