# Supplementary material for: Spatiotemporal Variation and the Role of Wildlife in Seasonal Water Quality Declines in the Chobe River, Botswana
Source: PLoS One. 2015 Oct 13;10(10):e0139936. doi: 10.1371/journal.pone.0139936 (PMC4603952; doi:10.1371/journal.pone.0139936)
Supplement: S1 Table — (DOCX) [file pone.0139936.s004.docx]

| **Data** | **Moran's I** | **E(X)** | **var(X)** | **Z-score** | **P-value** |
| --- | --- | --- | --- | --- | --- |
| Dry season *E. coli* | 0.227395 | -0.0011 | 5.9E-05 | 29.7514 | < 0.0001 |
| Dry season TSS | 0.24419 | -0.0012 | 7.1E-05 | 29.0642 | < 0.0001 |
| Wet season *E. coli* | 0.031631 | -0.0014 | 0.00002 | 7.42253 | < 0.0001 |
| Wet season TSS | 0.202886 | -0.0014 | 0.00011 | 19.7651 | < 0.0001 |
